# ^68^Ga-NEB PET/CT can be a new method for diagnosing chylous fistula: Case reports of a rare complication after breast cancer surgery

**DOI:** 10.1097/MD.0000000000021201

**Published:** 2020-07-17

**Authors:** Li Peng, Jialin Zhao, Feng Mao, Qiang Sun

**Affiliations:** Department of Breast Surgery, Peking Union Medical College Hospital, Chinese Academy of Medical Sciences and Peking Union Medical College, Beijing, China.

**Keywords:** ^68^Ga-NEB PET/CT, breast cancer surgery, chylous fistula, rare complication

## Abstract

**Rationale:**

We hypothesize that with the determination of lymph fistula location 3-dimensionally, application of appropriate pressure would promote fistula healing, and a secondary surgery may be avoided. ^68^Ga-labeled 1,4,7-triazacyclononane-N, N’, N”-triacetic acid (NOTA) conjugated with truncated Evan blue (NEB) forms a complex with serum albumin in the interstitial fluid after it is locally injected and allows rapid visualization of the lymphatic system.

**Patient concerns:**

A 44-year-old woman had a chief complaint of left nipple discharge. A 38-year-old woman came to the hospital after sensing a right breast mass.

**Diagnoses:**

The 2 patients were diagnosed with chylous fistula after breast cancer surgery based on the findings of a novel method, ^68^Ga-NOTA-Evans Blue (NEB) positron emission tomography/computed tomography.

**Interventions:**

We successfully obtained clear images to locate the fistula using ^68^Ga-NEB positron emission tomography/computed tomography (PET/CT) for both patients. The lymphatic vessels and lymph nodes could be clearly visualized owing to the ^68^Ga-NEB activity during PET/CT.

**Outcomes:**

Three-dimensional positioning to locate the fistula could direct the application of the pressure dressing and reduce drainage markedly.

**Lessons:**

^68^Ga-NEB PET/CT may be a new method for diagnosing chylous fistula and providing guidance for treatment.

## 1 Introduction

Chylous fistula can result from damage to the thoracic duct or its branches during various surgical procedures, such as neck or thoracic surgery.^[1,2]^ The fistula is an established yet rare complication after breast cancer surgery, and there are no specific methods to locate and treat it. To date, the reported incidence of chylous fistula after breast surgery is approximately 0.5%, and only a few cases after axillary lymph node dissection or mastectomy have been reported.^[3,4]^ In most cases, the fistula developed after axillary lymph node dissection, and <10% patients received sentinel lymph node biopsy (SLNB). It is very difficult to predict injury to the lymphatic trunks preoperatively or to identify this during surgery because of its rare occurrence during axillary or chest wall surgery; there are also no well-known risk factors. It is even more difficult to locate the injured branch after surgery. Approximately one-third of all patients undergo a reoperation.

^68^Ga-labeled 1,4,7-triazacyclononane-N,N’,N”-triaceticacid (NOTA) conjugated with truncated Evan blue (NEB) forms a complex with serum albumin in the interstitial fluid after local injection and allows rapid visualization of the lymphatic system.^[5,6]^ Herein, we present a case of chylous fistula after surgical treatment for breast cancer and try to find a new method for diagnosing and locating chylous fistula by positron emission tomography/computed tomography (PET/CT).

## 2 Case report

Preparation of NEB and 68Ga labeling was performed as described in previous publications.^[7,8]^ A Biograph 64 True Point TrueV PET/CT system (Siemens Medical Solutions, Erlangen, Germany) was used. 68Ga-NEB was injected subcutaneously into the bilateral first web spaces of the feet (0.5 mL, 37 MBq/foot), followed by massage of the injection sites. The patient was requested to walk after tracer injection. Images were acquired 5 to 20inutes after tracer injection. Whole-body images were acquired using a low-dose CT scan (120 kV, 35 mA, 3-mm layer, 512 × 512 matrix, 70 cm FOV). PET acquisition was performed (2 min/bed).

The study was approved by the Ethics Committee of Peking Union Medical College Hospital. Both patients provided written informed consent. Written informed consent for publication of this case report and the accompanying images was also obtained from the patients.

### 2.1 Case 1

**Figure 1:**
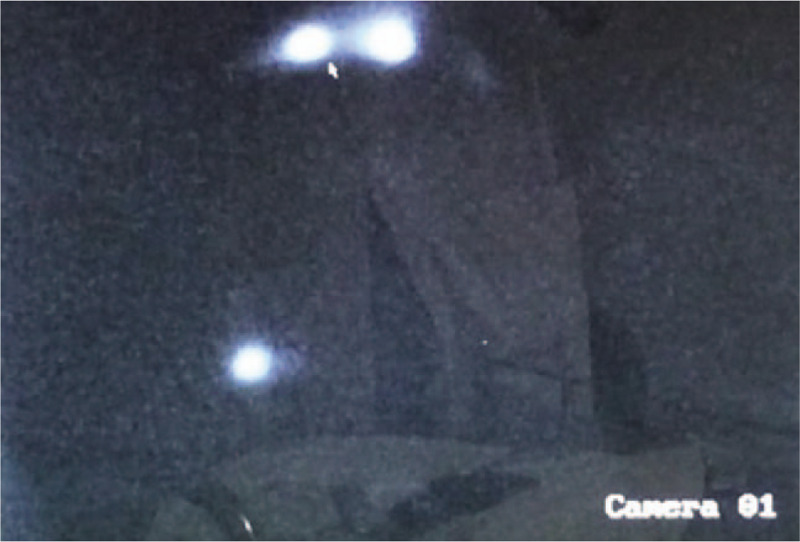
Arrow shows the injection point on the chest wall, and there was no clear chute on indocyanine green lymphangiography.

**Figure 2:**
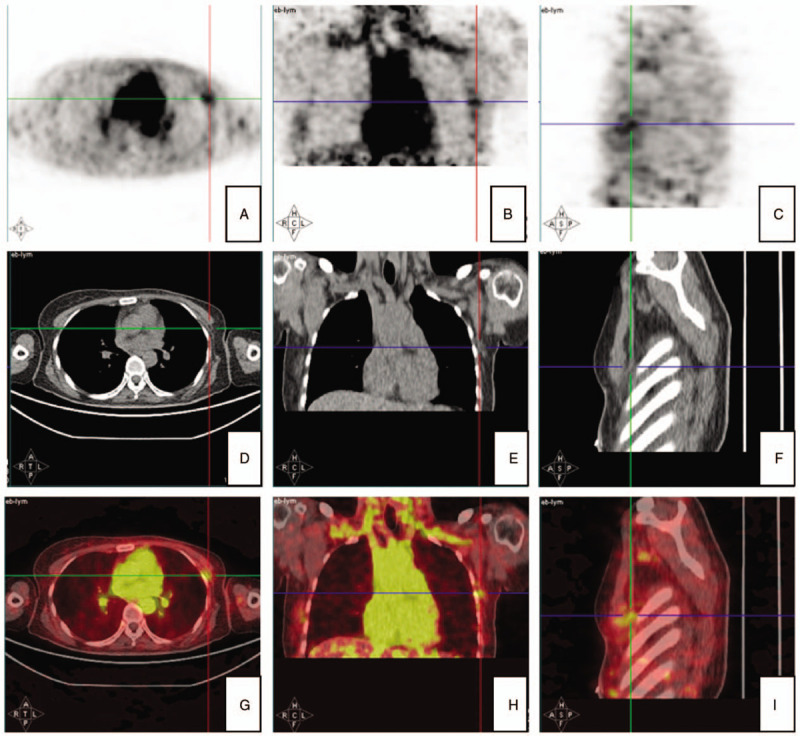
^68^Ga-NEB PET/CT enabled identification of the chylous fistula location on the inferior branch of the left subclavian lymph vessel after SLNB. Total general phase with delayed images indicated increasing tracer intensity at the left axilla from the (A) axial, (B) coronal, and (C) sagittal views. Lesion CT phase images showed the location of the fistula from (D) axial, (E) coronal, and (F) sagittal views. Lesion fusion phase images enabled fistula localization beyond the left subclavian vein, anterior to the left axillary vein from the (G) axial, (H) coronal, and (I) sagittal views.

A 44-year-old woman was admitted to our ward with a chief complaint of left nipple discharge. Biopsy indicated left breast atypical intraductal papilloma and partial intraductal papillary carcinoma. Simple mastectomy with SLNB was performed; the sentinel lymph node was not involved (0/2). On postoperative day (POD) 7, a large amount of milky white turbid liquid exuded from the surgical incision and was positive for Sudan red staining. Daily drainage from the drainage tube was 200 to 300 mL. Indocyanine green lymphangiography showed no clear chute (Fig. 1). ^68^Ga-NEB PET/CT scan indicated a lymphatic leakage on the inferior branch of the left subclavian lymph vessel in the left axilla (Fig. 2). Pressure dressing in the left axilla, systemic anti-infective therapy, and a low-fat diet were given. The patient's wound healed well, and adjuvant treatments were not delayed. The patient received endocrine therapy and was noted to be doing well 3 years postoperatively.

### 2.2 Case 2

**Figure 3:**
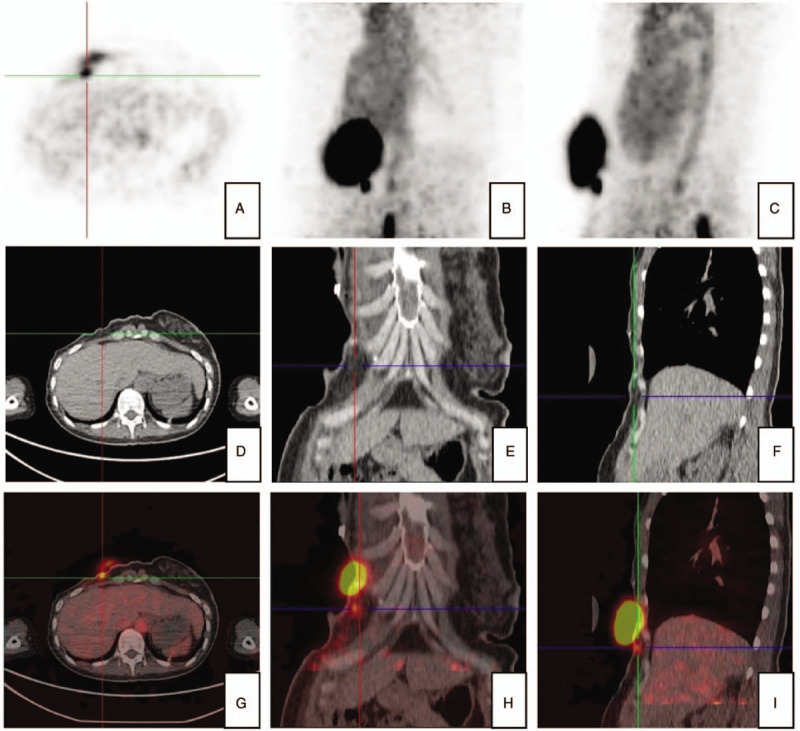
^68^Ga-NEB PET/CT enabled identification of the chylous fistula location. Overall delayed phase images indicated increasing tracer intensity from the (A) axial, (B) left anterior oblique, and (C) sagittal views. Lesion CT phase images showed the location of the fistula from the (D) axial, (E) left anterior oblique, and (F) sagittal views. Lesion fusion phase images enabled fistula localization at the level of the fifth front rib vein from the (G) axial, (H) left anterior oblique, and (I) sagittal views.

A 38-year-old woman came to our hospital after sensing a right breast mass. Modified radical mastectomy was conducted, and postoperative pathology was triple negative invasive ductal carcinoma without lymph node involvement. About 20-mL yellow exudate was observed on the dressing on POD 2 and maintained about 50 to 100 mL each day. ^68^Ga-NEB PET/CT scan indicated a lymphatic leakage (Fig. 3). Pressure dressing toward the left chest and low-fat diet were given. Drainage reduced gradually daily, and the patient was discharged 12 days after the examination. Then, she received 4 cycles of chemotherapy as expected with no additional complications and did not experience recurrence 2 years postoperatively.

Both patients were diagnosed with lymphatic fistula preliminarily within 1 week after operation. The amount of the drainage was <300 mL each day, so we speculated that there was a small damage to the lymphatic branch. We confirmed our initial speculations by ^68^Ga-NEB PET/CT. Total general phase images showed increasing tracer intensity, and the lesion fusion phase showed the location of the chylous fistula. In both patients, the lymphatic fistula had a nodular appearance. We could even see the site of the fistula and leaking fluid labeled by ^68^Ga-NEB in the same image, and the labeled leaking fluid formed a drainage pool in front of the chest wall.

## 3 Discussion

Chylous fistula rarely occurs after axillary surgery because the axilla is anatomically remote from the thoracic duct. The reported incidence of chylous fistula after neck surgery is 1% to 3%,^[2]^ and its incidence after surgery for breast cancer is around 0.5%,^[4]^ with 75% to 92% occurrence on the left side.^[9]^ Its occurrence pattern in the thoracic duct was varied, or it may have continuous drainage over 500 to 600 mL per day without improvement.^[4,9]^ Some lymph vessels may be injured after breast surgery, which include the subclavian branch, descending intercostal branch (at the fifth intercostal level), abnormal branch between the thoracodorsal and long thoracic nerves in front of the subscapularis muscle, and various sites in the thoracic duct.^[10]^

We have summarized previously published cases of chylous leakage after breast surgery. Most of them occurred after axillary lymph node biopsy, and <10% of patients had undergone SLNB. Almost all chylous fistula cases occurred on the left side. However, Cong et al^[11]^ reported 2 cases that occurred on the other side. It is inferred that the thoracic duct occasionally divides at its upper part into 2 branches, with the left branch ending in the usual manner, whereas the right branch opens into the right subclavian vein connected with the right lymphatic duct. Communicating branches between the lymph ducts distributed in the abdominal wall and in the abdominal cavity may drain lymph fluids to the superficial lymph net. Trauma to the superficial lymph network distributed in the chest and abdominal wall may have been responsible for the leakage.^[12]^ About one-third of all patients underwent reoperation, and Oba et al^[13]^ supposed that surgical treatment should be considered if patients had >700 mL of chylous leakage per day. We can infer that if we can locate the fistula 3-dimensionally, maintaining accurate pressure can promote healing of the fistula and probably avoid secondary surgery.

Predicting injury to the lymphatic trunks preoperatively or its recognition intraoperatively is very challenging because chylous fistula rarely occurs after axillary surgery, and well-known risk factors are not yet established. Moreover, locating the injured branch after breast surgery is even more difficult.^[14]^ Thus, determining the location of the chylous fistula is important for its diagnosis and control. Thang et al^[15]^ described the value of hybrid single-photon emission computed tomography/computed tomography lymphoscintigraphy, which provided better localization of the postoperative chyloma leakage site. Since PET/CT has been widely performed in breast cancer patients, we speculate that PET/CT might help locate the fistula, as it could mark the lymph nodes.^[16]^ In these 2 cases, we successfully obtained clear images and located the fistula using ^68^Ga-NEB PET/CT. ^68^Ga-NEB activity can clearly help visualize lymphatic vessels and lymph nodes by PET/CT. The preparation of NEB and ^68^Ga labeling were performed as described in previous publications.^[5,6]^^68^Ga-NEB PET/CT requires significantly less waiting time after tracer injection and provides more clinically important information compared to ^99m^Tc-SC lymphoscintigraphy.^[7,8]^ This is conceivable because ^68^Ga-NEB PET/CT images are three-dimensional (3D) images and involve CT correlation, whereas the traditional ^99m^Tc-SC lymphoscintigraphy acquires only static images without CT correlation. The 3D positioning to determine the fistula could direct the pressure dressing and reduce the drainage distinctly. Majority of chylous fistula cases respond to conservative management.^[12]^ Accurate pressure can promote fistula healing and possibly avoid secondary surgery. In conclusion, ^68^Ga-NEB PET/CT may be a new method for diagnosing chylous fistula and provide guidance for treatment.

## Acknowledgments

The authors thank Jingjing Zhang for her assistance with the figures.

## Author contributions

Li Peng: Conceptualization; Data curation; Formal analysis; Methodology; Visualization; Writing – original draft.

Jialin Zhao: Investigation; Methodology; Project administration Writing – original draft.

Feng Mao: Resources; Software.

Qiang Sun: Supervision; Validation; Writing – review & editing.

Li Peng and Jialin Zhao have contributed equally to this work.
